# Cardiovascular and Renal Outcomes Following Acute Kidney Injury in Pregnancy: A Systematic Review and Meta‐Analysis

**DOI:** 10.1111/1471-0528.18352

**Published:** 2025-09-24

**Authors:** Deepthika Jeyaraman, Dimuth P. Peiris, Mark Lambie, Kate Bramham, Richard Fish, Haia Alahmdi, Mamas A. Mamas, Pensée Wu

**Affiliations:** ^1^ Academic Department of Obstetrics and Gynaecology University Hospital of North Midlands Stoke‐on‐Trent UK; ^2^ Education Training & Research Unit Ministry of Health Colombo Sri Lanka; ^3^ Department of Nephrology University Hospitals of North Midlands Stoke‐on‐Trent UK; ^4^ School of Medicine Keele University Keele UK; ^5^ Department of Women and Children's Health King's College London London UK; ^6^ Royal College of Surgeons in Ireland Medical University of Bahrain Al Sayh Muharraq Governorate Bahrain; ^7^ Keele Cardiovascular Research Group, Centre for Prognosis Research Keele University Keele UK; ^8^ Department of Obstetrics and Gynaecology, College of Medicine National Cheng Kung University Tainan Taiwan

**Keywords:** acute kidney injury, cardiovascular, outcomes, pregnancy, renal

## Abstract

**Background:**

Acute kidney injury (AKI) in pregnancy is associated with adverse maternal and foetal outcomes. However, there is limited evidence regarding cardiac and renal outcomes associated with AKI in pregnancy.

**Objective:**

To quantify and perform a meta‐analysis of the risk of adverse cardiovascular and renal outcomes following AKI in pregnancy.

**Search Strategy:**

A systematic search of MEDLINE, Cochrane Library and EMBASE from inception until 23 January 2024.

**Selection Criteria:**

Studies investigating adverse cardiovascular and renal outcomes in pregnant patients with AKI.

**Data Collection and Analysis:**

Two reviewers independently performed screening, data extraction and quality assessment. A random‐effects model was used to estimate risk.

**Main Results:**

A total of 17 studies were included with 50 285 836 pregnant women, of which 36 806 women were affected by AKI. Our evidence synthesis showed that AKI in pregnancy is associated with a 52‐fold increase in the risk of composite adverse renal outcomes (OR 52.37; 95% CI 4.67–587.63), a 23‐fold increase in the risk of heart failure (OR 22.55; 95% CI 4.39–115.71) and stroke (OR 22.92; 95% CI 2.32–226.65), as well as a 9.3‐fold and 3.9‐fold increased risk of maternal mortality (OR 9.26; 95% CI 2.53–33.96) and intensive care unit admission (OR 3.86; 95% CI 1.93–7.71), respectively.

**Conclusions:**

The study shows that AKI in pregnancy is associated with adverse cardiovascular and renal outcomes. Careful monitoring and follow‐up of patients with AKI in pregnancy may enable earlier detection and management of some adverse cardiovascular and renal outcomes.

## 1 Introduction

Acute kidney injury (AKI) occurs in approximately 2% of pregnancies [1], and is associated with significant maternal morbidity. It is becoming more prevalent due to increasing age and maternal comorbidities [2]. Despite the increase in the prevalence of AKI in pregnancy, there remains a paucity of research in this field.

AKI in pregnancy is considered a sudden deterioration in renal function during pregnancy [3]. Outside pregnancy, AKI is defined by either a fall in urine output and/or a rise in serum creatinine [3]. Due to the physiological reduction in serum creatinine in pregnancy and the dynamic nature of creatinine in pregnancy, there is currently no agreed and robust definition for AKI in pregnancy and no existing AKI criteria are validated for use in pregnancy [2].

The association between AKI in pregnancy and adverse maternal and foetal outcomes has been previously described in the literature. These include maternal death, intensive care unit admission, haemorrhage, stillbirth, preterm birth and low birth weight [4]. AKI has also been associated with some adverse cardiovascular and renal outcomes. For example, cardiovascular events, dialysis, chronic kidney disease and end‐stage renal failure [1, 5]. However, adverse cardiovascular and renal outcomes following AKI in pregnancy have not been systematically evaluated. To this end, we aimed to quantify the risk of adverse cardiovascular and renal outcomes following AKI in pregnancy and to determine the variation in AKI definition.

## 2 Methods

### 2.1 Data Sources and Searches

The protocol was registered in PROSPERO (CRD42022378572). We searched MEDLINE and EMBASE from inception for studies from inception to 21 June 2022, with an updated search on 23 January 2024 with the support of a clinical effectiveness librarian. The detailed search strategy is shown in Table S1. We also manually searched the reference lists of the included studies and relevant reviews to identify potential additional studies for inclusion.

### 2.2 Eligibility Criteria

We selected studies on cardiovascular and renal outcomes in pregnancies with and without AKI published in the English language. There was no restriction on the definition of AKI. The cardiovascular and renal outcomes of interest were maternal mortality, hypertension, gestational hypertension, heart failure, stroke, thrombotic microangiopathy, partial renal recovery, renal insufficiency, chronic kidney disease, renal replacement therapy and intensive care unit admissions (Table S2). The studies included had at least two groups, namely pregnant mothers with and without AKI. There was no restriction based on cohort type, study design, or duration of follow‐up in the study selection. Studies which had fewer than 10 participants with AKI, and studies which focused on a selected population (i.e., renal transplantation and chronic kidney disease) were excluded.

### 2.3 Study Selection and Data Extraction

Using the software Rayyan [6], double title and abstract screening were performed (H.A. and D.J.), followed by double full text screening (D.J. and D.P.P.). Any disagreement was resolved through discussion. Any remaining conflicts were resolved by discussion with a third reviewer (P.W.) who made the final decision. Data extraction was performed by two reviewers (D.J. and D.P.P.) independently.

### 2.4 Study Quality Assessment

We used the Newcastle‐Ottawa Quality Assessment Scale for cohort studies and case–control studies for quality assessment. The study quality was assessed using the following criteria: representativeness of the exposed cohort, comparability of the exposed cohort to the non‐exposed cohort, ascertainment of AKI exposure, exclusion of participants with the outcome of interest at the start of the study, ascertainment of the outcome, duration of follow‐up and loss to follow‐up. Scores of 7 to 9, 4 to 6 and less than 4 were classified as having a low, moderate, or high risk of bias, respectively [7, 8].

### 2.5 Data Synthesis and Analysis

RevMan version 5.4 (Nordic Cochrane Centre) was used to conduct random‐effects meta‐analysis using the inverse variance method for pooling odds ratios (ORs). The random effects were used to account for heterogeneity as the studies were from various settings and populations. We used the adjusted odds ratios from the primary studies when available; otherwise, unadjusted odds ratios were calculated using the raw data. We assessed the statistical heterogeneity using the *I* ^2^ statistic, where *I* ^2^ values between 30% and 60% were considered a moderate level of heterogeneity. We performed leave‐one‐out analysis to identify studies that contributed to a high degree of heterogeneity if there was greater than a moderate degree of heterogeneity. Funnel plots were used to assess for publication bias where there are > 10 studies and little evidence of heterogeneity. We conducted subgroup analysis considering different definitions of AKI in outcomes where enough studies were available. We also stratified the studies into pre‐eclampsia (> 50% study population) versus no pre‐eclampsia subgroups. The Comprehensive Meta‐Analysis prediction intervals program was used to calculate prediction intervals (PI) [9].

Patients were not involved in the development of the research. A core outcome set has not been used. The first author (D.J.) is funded by the NIHR Integrated Academic Training Programme.

## 3 Results

### 3.1 Description of Studies Included in Analysis

**FIGURE 1:**
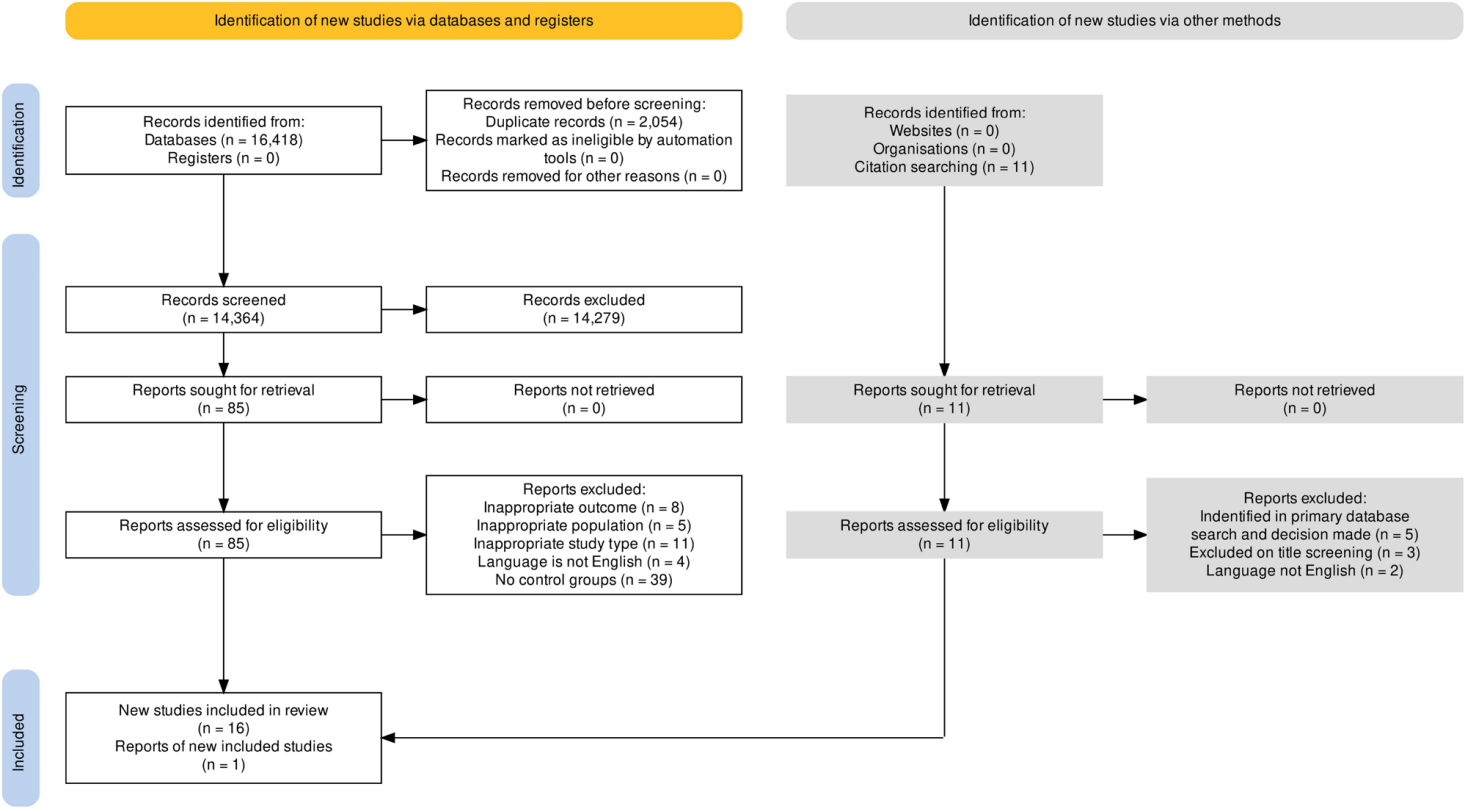
Flow diagram of study inclusion.

The search identified 15 474 titles and abstracts. Following deduplication and screening, 17 studies were included in this analysis (Figure 1). We included 50 285 836 pregnant women, with 36 806 women affected by AKI. Table S3 shows the details of the included studies. Out of the 17 studies, 9 studies focused on women with pre‐eclampsia (*n* = 3256 women); out of this group, 853 women had AKI. The length of follow‐up ranged from 6 days to 2 years following delivery. Five studies were conducted in China two were in the United States, and one each from the following countries: South Africa, Spain, Pakistan, Malawi, India, Tunisia, Morocco, the United Kingdom, Canada and Turkey.

Two studies had overlapping populations but investigated different outcomes, except for maternal mortality [5, 10]. Therefore, we only included the study with the largest number of participants in the total study population and used the data from this study in the meta‐analysis for the maternal mortality outcome [10].

### 3.2 Quality Assessment of Included Studies

The study quality was evaluated using the Newcastle‐Ottawa Quality Assessment Scale for cohort and case–control studies as shown in Table S4. Sixteen studies had reliable ascertainment of AKI in pregnancy, ascertainment of outcomes, and outcomes of interest not being present at the start of pregnancy. Twelve studies had a reliable method for selection of the non‐AKI population, while eight studies had a representative population of AKI in pregnancy, which primarily included women of childbearing age who required hospitalisation for AKI in pregnancy. Overall, 15 out of the 17 included studies were of low or moderate risk of bias.

### 3.3 AKI in Pregnancy and Adverse Renal Outcomes

**FIGURE 2:**
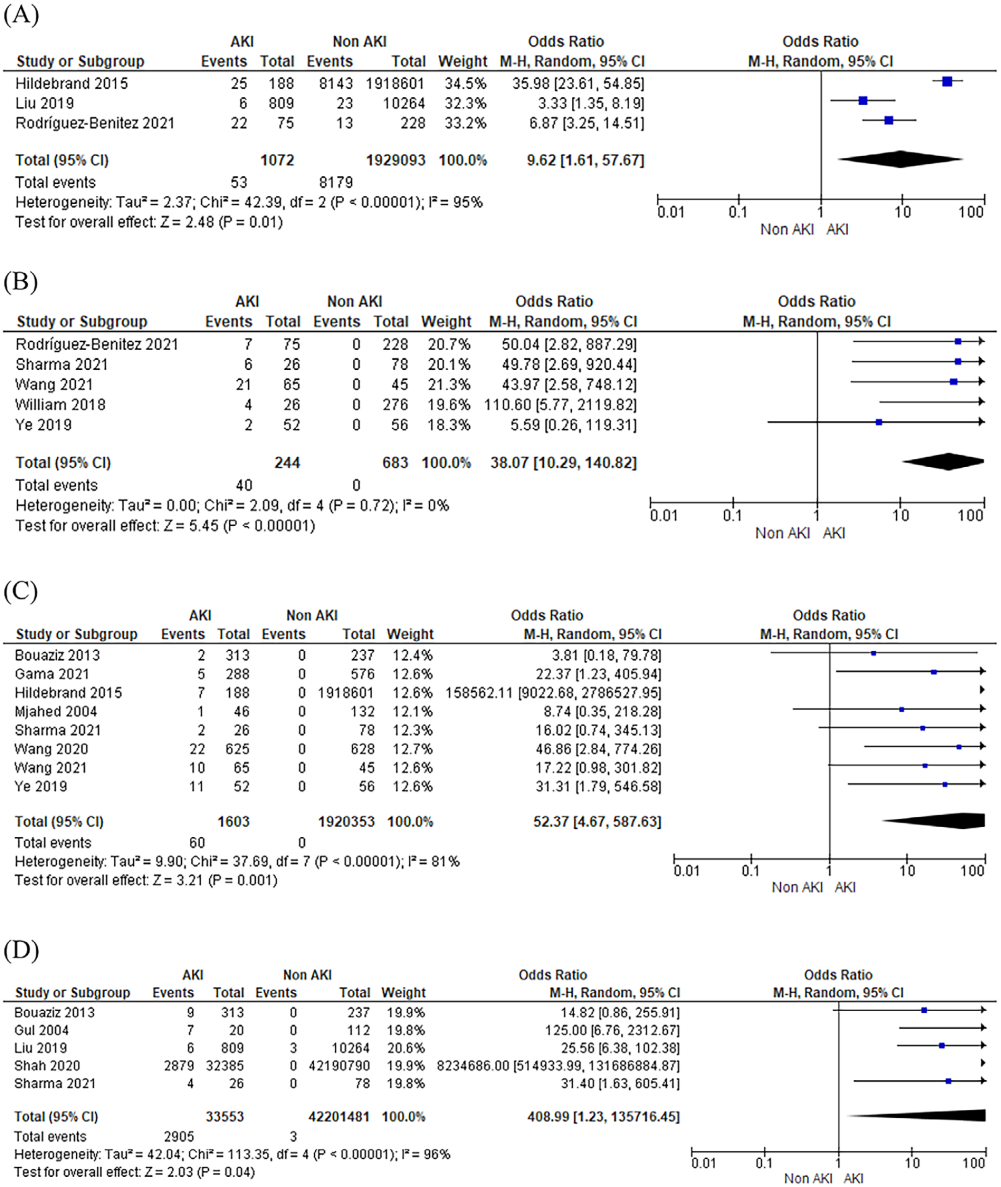
Risk of adverse renal outcomes with acute kidney injury in pregnancy. (A) Thrombotic microangiopathy. (B) Prolonged renal insufficiency, including partial renal recovery. (C) Composite of chronic kidney disease and renal replacement therapy. (D) Dialysis.

AKI in pregnancy was associated with a 9.6‐fold increase in risk of thrombotic microangiopathy (OR 9.62; 95% CI 1.61–57.67; 95% PI 0–72 782 359 066.21; *I* ^2^ = 95%; 3 studies), with no causative link (Figure 2A). AKI was also associated with a 38‐fold increase in risk of prolonged renal insufficiency (OR 38.07; 95% CI 10.29–140.82; 95% PI 10.29–140.82; *I* ^2^ = 0%; 5 studies), compared with no AKI in pregnancy (Figure 2B). Prolonged renal insufficiency occurred over several years, as shown by the duration of study follow‐up in Table S3. When considering chronic kidney disease and renal replacement therapy together, women with AKI in pregnancy had a 52‐fold increase in the risk of composite adverse renal outcomes (OR 52.37; 95% CI 4.67–587.63; 95% PI 0.01–204 413.70; *I* ^2^ = 81%; 8 studies) compared to women without AKI in pregnancy (Figure 2C). The need for dialysis was 409 times higher in women with AKI in pregnancy (OR 408.99; 95% CI 1.23–135 716.45; 95% PI 0–2 908 674 832 174.70; *I* ^2^ = 96%; 5 studies) compared to those without AKI in pregnancy, but with a wide confidence interval and high heterogeneity among the studies (Figure 2D).

### 3.4 AKI in Pregnancy and Adverse Cardiovascular Outcomes

**FIGURE 3:**
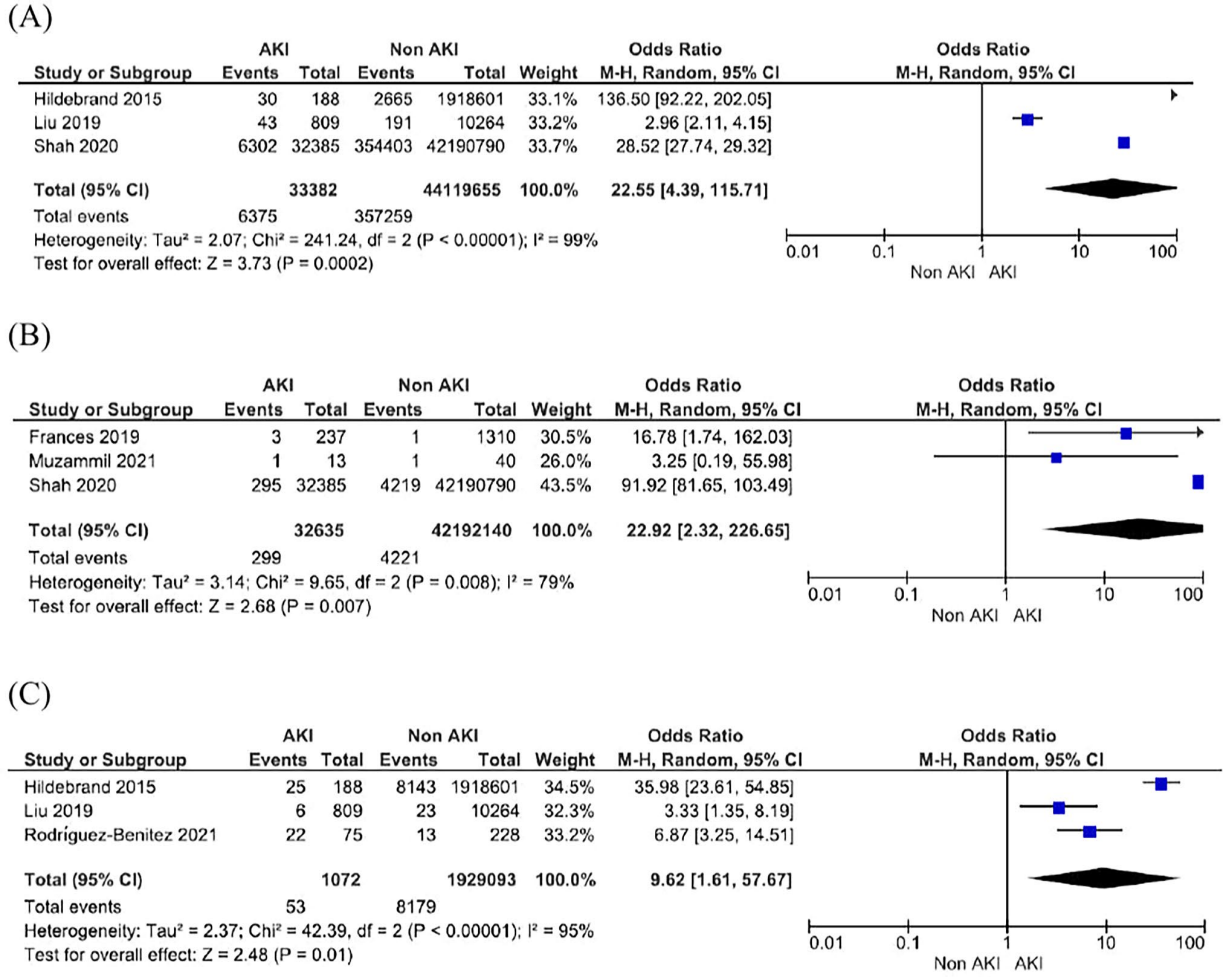
Risk of adverse cardiovascular outcomes with acute kidney injury in pregnancy. (A) Heart failure. (B) Stroke. (C) Peripheral vascular disease.

AKI in pregnancy was associated with a 23‐fold increase in risk of stroke (OR 22.92; 95% CI 2.32–226.65; 95% PI 0–11 883 161 737 018.60; *I* ^2^ = 79%; 3 studies), a 22‐fold increase in risk of heart failure (OR 22.55; 95% CI 4.39–115.71; 95% PI 0–33 958 976 023.23; *I* ^2^ = 99%; 3 studies), and a 9.6‐fold increase in risk of peripheral vascular disease (OR 9.62, 95% CI 1.61–57.67, 95% PI 0–72 782 359 066.21; *I* ^2^ = 95%; 3 studies) compared to pregnant women without AKI (Figure 3). However, high heterogeneity was observed between these studies. AKI in pregnancy was not associated with hypertension (OR 1.22; 95% CI 0.44–3.40; 95% PI 0–279 437.47; *I* ^2^ = 88%; 3 studies) or gestational hypertension (OR 1.00; 95% CI 0.33–3.02; 95% PI not available as < 3 studies; *I* ^2^ = 78%; 2 studies) as shown in Figure S1.

### 3.5 AKI in Pregnancy and Intensive Care Unit Admission or Maternal Mortality

**FIGURE 4:**
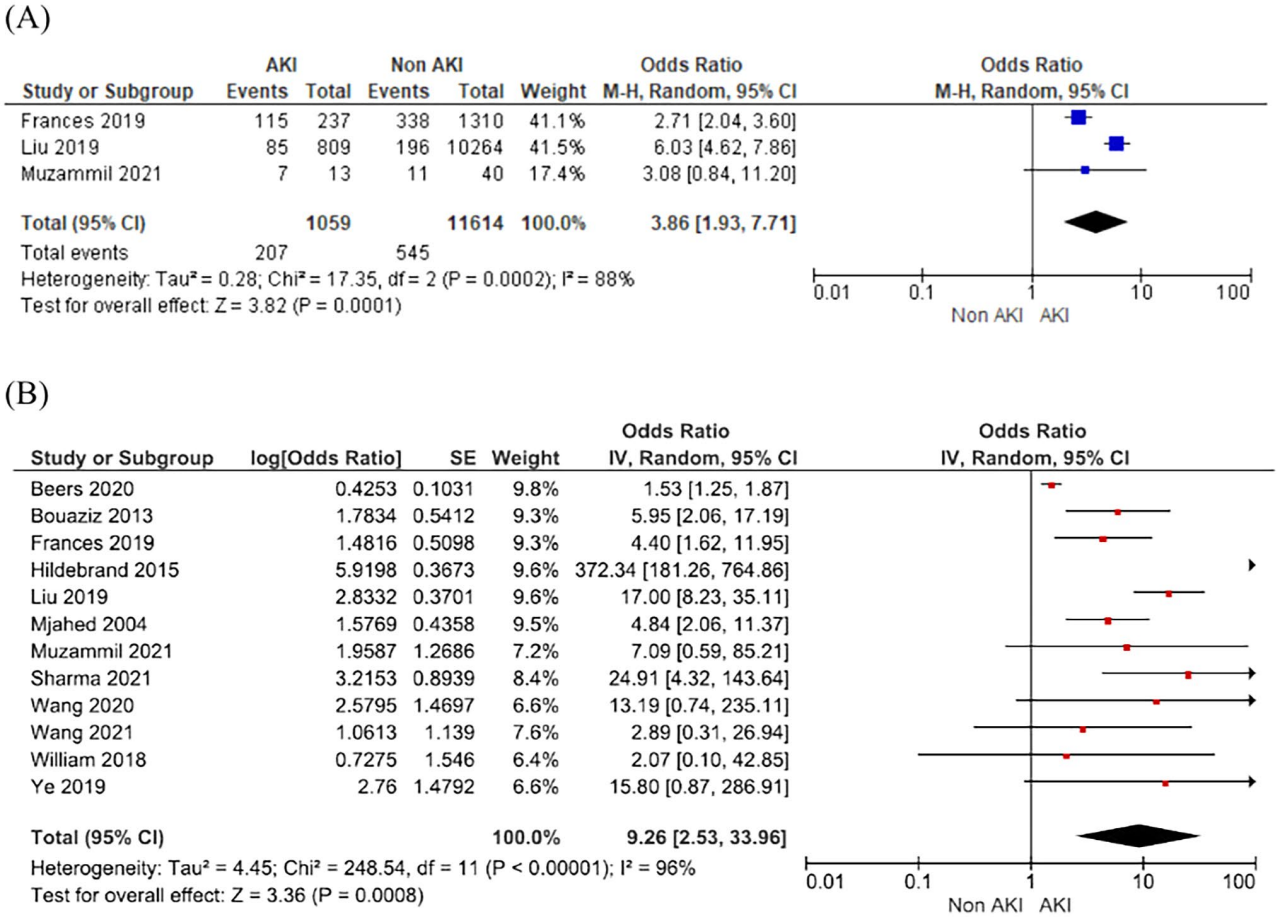
Acute kidney injury in pregnancy and risk of (A) intensive care unit admission and (B) maternal mortality.

There is a 3.9‐fold increase in the risk of intensive care unit admission (OR 3.86; 95% CI 1.93–7.71; 95% PI 0–12 491.93; *I* ^2^ = 88%; 3 studies) and 9.3‐fold increase in maternal mortality (OR 9.26; 95% CI 2.52–33.96; 95% PI 0.07–1277.46; *I* ^2^ = 96%; 12 studies) in women with AKI in pregnancy compared to women without AKI in pregnancy (Figure 4).

### 3.6 Additional Analyses

As we had observed a difference in the definition of AKI between the included studies (Table S3), we performed a subgroup analysis between AKI defined according to the Kidney Disease: Improving Global Outcomes (KDIGO) classification and arbitrary serum creatinine value (Table S5). We were only able to analyse outcomes with a sufficient number of studies. There was a higher risk of composite adverse renal outcomes (chronic kidney disease and renal replacement therapy) from AKI when assessed using KDIGO classification compared to arbitrary serum creatinine levels (KDIGO classification: OR 21.15; 95% CI 4.91–91.06; *I* ^2^ = 0%; 4 studies versus arbitrary serum creatinine level: OR 12.69; 95% CI 2.24–72.01; *I* ^2^ = 0%; 3 studies). However, there was a lower risk of maternal mortality in women with AKI defined using the KDIGO classification (OR 6.10; 95% CI 2.96–12.57; *I* ^2^ = 0%; 6 studies), compared with women with AKI defined by an arbitrary serum creatinine level (OR 8.55; 95% CI 4.12–17.73; *I* ^2^ = 47%; 4 studies).

As pre‐eclampsia can adversely affect renal function in pregnancy, we assessed women with and without pre‐eclampsia in a subgroup analysis (Table S6). AKI increases the risk of composite adverse renal outcomes by 13‐fold (OR 12.83; 95% CI 3.35–49.13; *I* ^2^ = 0%; 5 studies) in women with pre‐eclampsia. Women with pre‐eclampsia and AKI had a 5.6‐fold increased risk of maternal mortality (OR 5.63; 95% CI 3.45–9.18; *I* ^2^ = 0%; 8 studies) and a 2.7‐fold increase in the risk of ICU admission (OR 2.73; 95% CI 2.07–3.60; *I* ^2^ = 0%; 2 studies) compared to women with pre‐eclampsia and no AKI. However, AKI in pregnancy increased the risk of adverse cardiovascular and renal outcomes to a greater extent in women without pre‐eclampsia compared to women with pre‐eclampsia.

We performed a subgroup analysis based on study location due to the diverse geography between the included studies (Table S7). None of the included studies were conducted in South America. In Asia, there was an increased risk of maternal mortality, ICU admission, heart failure, thrombotic microangiopathy, dialysis, partial renal recovery, or renal insufficiency and composite adverse renal outcomes. In Africa, there was an increased risk of maternal mortality, ICU admission, stroke and partial renal recovery or renal insufficiency, but there was no significant increase in composite adverse renal outcomes or need for dialysis. In Europe, there was an increased risk of composite adverse renal outcomes and thrombotic microangiopathy. Finally, in North America, there was an increased risk of maternal mortality, heart failure, stroke, thrombotic microangiopathy, dialysis and composite adverse renal outcomes.

Leave‐out analyses were performed for all outcomes except gestational hypertension due to a low number of studies (*n* = 2). We did not identify the source of heterogeneity. Funnel plots were not performed due to none of the outcomes having more than 10 studies with little evidence of heterogeneity.

## 4 Discussion

### 4.1 Main Findings

Our systematic review and meta‐analysis of 17 studies included over 50 million women, with over 36 000 women affected by AKI. Our evidence synthesis showed that AKI in pregnancy is associated with a 52‐fold increase in risk of composite adverse renal outcomes, a 10‐fold risk of maternal mortality and a 4‐fold increased risk of intensive care unit admission. However, AKI in pregnancy is not associated with an increase in risk of hypertension. Subgroup analysis showed there was a higher risk of composite adverse renal outcomes when AKI was defined using the KDIGO classification compared with using an arbitrary serum creatinine level. To our knowledge, this meta‐analysis is the first to assess the impact of AKI in pregnancy on cardiovascular and renal outcomes.

### 4.2 Interpretation

The mechanisms underlying the observed associations in our meta‐analysis remain to be elucidated as no causative link has been demonstrated in our study. The relationship between AKI and some of the adverse cardiovascular and renal outcomes studied, in particular ICU admission and maternal mortality, may be an association due to the severity of the underlying issue or obstetric complication rather than causative. AKI in pregnancy is associated with localised and systemic inflammatory response through increased pro‐inflammatory mediators such as IFNα, IL‐2 and IL‐6 [11]. Inflammatory markers are known to be involved in the pathogenesis underpinning atherosclerosis and its complications [12]. Furthermore, endothelial dysfunction and interstitial inflammation associated with raised pro‐inflammatory markers can cause fibrosis that contributes to the development of chronic kidney disease [13]. Therefore, inflammation associated with AKI may contribute to long‐term adverse cardiovascular and renal outcomes. However, shared risk factors between cardiovascular disease, chronic kidney disease, AKI and pregnancy create a lack of clarity in the underlying mechanism.

In our subgroup analysis of AKI definitions, we found AKI defined using the KDIGO classification was associated with a higher risk of composite adverse renal outcome and a lower risk of maternal mortality compared with those defined using an arbitrary creatinine level. Serum creatinine levels vary based on multiple levels such as age, sex, dietary intake and medications consumed [14], while the KDIGO AKI classification system uses relative serum creatinine or urine output changes to diagnose AKI rather than a threshold set by an arbitrary serum creatinine value. As serum creatinine reduces in pregnancy, a mild increase in creatinine level may be more clinically significant in pregnancy compared to outside pregnancy. The KDIGO guidelines incorporate urine output as part of AKI diagnosis in the non‐pregnant state, but hydration and haemodynamic status, which influence urine output, are altered in pregnancy. Unfortunately, there is no current agreed standardised classification for AKI in pregnancy and none of the existing classifications have been validated for pregnancy. Therefore, the results of our meta‐analysis are limited by the heterogeneity in AKI definitions [15, 16]. Of note, there is also heterogeneity in the definition of chronic kidney disease in pregnancy [17].

Our subgroup analysis on women with pre‐eclampsia suggests that AKI increases the risk of maternal mortality further to that already observed in pregnancies affected by pre‐eclampsia [18, 19]. Therefore, AKI may worsen the adverse physiological impact of pre‐eclampsia. As hypertensive disorders of pregnancy, which include pre‐eclampsia, account for the most common cause of AKI [20], it is important to monitor this high‐risk patient population. Nevertheless, for women without pre‐eclampsia, AKI in pregnancy increased the risk of adverse cardiovascular and renal outcomes to a greater extent than for women with pre‐eclampsia. This is because women with pre‐eclampsia have a higher baseline risk of adverse outcomes compared to women without pre‐eclampsia. Current evidence suggests that AKI in pregnancy is a useful surrogate marker for the severity of pre‐eclampsia in pregnancy [21]. Due to the lack of clear evidence indicating causation, it is possible that severe pre‐eclampsia, which is usually associated with AKI in pregnancy, could be an underlying mechanism of increased risk of adverse cardiovascular and renal outcomes.

### 4.3 Strengths and Limitations

The main strength of our study is our large sample size and a comprehensive search which included all relevant studies to date. Our robust methodology included a librarian to verify the search strategy and separate reviewers to conduct independent screening and data extraction. Limitations include publication bias and lack of inclusion of non‐English and grey literature. As a proportion of included studies were retrospective, we had limited control over the quality of data collected. There could have been incomplete, inaccurate, or inconsistent historical data which could have affected whether the case and control groups were ascribed correctly. The high heterogeneity between studies may have arisen due to differences in the study population, research methodology, study period, country where the study was conducted, and inherent differences between the studies. Some outcomes, including stroke, heart failure and dialysis, had a small sample size which can result in wide confidence intervals and thus limit the clinical usefulness of the results. Furthermore, it is worth noting that the inverse variance weighting model used in this study has many limitations compared with the generalised linear mixed model, especially when there are small sample sizes. As our meta‐analysis includes studies from different geographical areas with different health care systems, there may be healthcare inequalities that may have affected our findings. For example, inequitable access to ICU care could have impacted ICU admission, rather than illness severity. Finally, some studies had included a proportion of pregnant women with chronic kidney disease, while the majority of studies did not report whether women with chronic kidney disease were excluded. This could lead to potential overestimation of the level of risk quantified in our meta‐analysis.

## 5 Conclusion

Our study highlights the importance of AKI in pregnancy as a risk factor for significant future adverse cardiovascular and renal outcomes. As a common renal problem in pregnancy, it is crucial to ensure women who sustain AKI in pregnancy are followed up and monitored appropriately. In the future, researchers need to consider ways to standardise the definition and measurement of AKI in pregnant women, as a robust definition for AKI in pregnancy is still not available. There is an urgent need for high‐quality primary studies as there are limited studies focusing on cardiovascular and renal outcomes with a validated measurement for AKI in pregnancy. Furthermore, studies to elucidate the potential underlying mechanisms of these associations will benefit management of women with AKI in pregnancy. Further studies should include information on AKI severity and gestational age when AKI developed, as the majority of the studies did not include these details.

In conclusion, our study has shown that AKI is associated with long‐term adverse cardiovascular and renal outcomes which increase the risk burden for this population. The study, with its limitations, highlights the potential implications for patient care via surveillance, early detection of risk factors and early management to optimise risk factors of adverse cardiovascular and renal outcomes.

## Author Contributions

The systematic review was conceptualised following a literature review and discussion among P.W., M.L., K.B., R.F., H.A., D.P.P. and D.J. Study screening and data extraction were performed by H.A., D.P.P. and D.J. All team members were involved in the protocol writing, data analysis and drafting of the manuscript and its critical revision. The corresponding author (D.J.) confirms that all authors gave final approval for the version to be published and agree to be accountable for all aspects of the work.

## Ethics Statement

The authors have nothing to report.

## Conflicts of Interest

The authors declare no conflicts of interest.

## Supporting information


**Figure S1:** Risk of (A) hypertension or (B) gestational hypertension with acute kidney injury in pregnancy.
**Table S1:** Search strategy.
**Table S2:** Outcome of interest and definitions used in this study.
**Table S3:** Study design and participant characteristics.
**Table S4:** Study quality assessment.
**Table S5:** Subgroup analysis of outcomes of AKI in pregnancy based on the definition.
**Table S6:** Subgroup analysis of outcomes of AKI in pregnancy based on the presence of pre‐eclampsia.
**Table S7:** Subgroup analysis based on study location.

## Data Availability

Data sharing is not applicable to this article as no new data were created or analysed in this study.
