# Supplementary material for: Cardiovascular and Renal Outcomes Following Acute Kidney Injury in Pregnancy: A Systematic Review and Meta‐Analysis
Source: BJOG. 2025 Sep 24;133(1):44–51. doi: 10.1111/1471-0528.18352 (PMC12676191; doi:10.1111/1471-0528.18352)
Supplement: Supplementary file 1 — Figure S1: Risk of (A) hypertension or (B) gestational hypertension with acute kidney injury in pregnancy. Table S1: Search strategy. Table S2: Outcome of interest and definitions used in this study. Table S3: Study design and participant characteristics. Table S4: Study quality assessment. Table S5: Subgroup analysis of outcomes of AKI in pregnancy based on the definition. Table S6: Subgroup analysis of outcomes of AKI in pregnancy based on the presence of pre‐eclampsia. Table S7: Subgroup analysis based on study location. [file BJO-133-44-s001.docx]

**Supplementary Materials**

**Table S1. Search Strategy**

| **Ovid MEDLINE Search Strategy** | | |
| --- | --- | --- |
| 1 | pregnancy/ | 953132 |
| 2 | conception/ | 16147 |
| 3 | childbirth/ | 12335 |
| 4 | fetus/ | 81627 |
| 5 | newborn/ | 651331 |
| 6 | (pregnan$ or gestation$ or (new adj5 born) or labo$ or fetal$).tw. | 1513889 |
| 7 | 1 or 2 or 3 or 4 or 5 or 6 | 2342872 |
| 8 | clinical trial/ | 535421 |
| 9 | exp cohort analysis/ | 2363327 |
| 10 | exp case control study/ | 1331404 |
| 11 | exp prospective study/ | 631371 |
| 12 | retrospective study/ | 1037835 |
| 13 | exp longitudinal study/ | 158895 |
| 14 | exp follow up/ | 0 |
| 15 | (cohort$ or (case$ adj5 control$) or (longitudinal$ adj5 stud$) or (follow$ adj5 stud$)).tw. | 1214565 |
| 16 | (prospective$ adj5 stud$).mp. or (retrospective$ adj5 stud$).tw. [mp=title, abstract, original title, name of substance word, subject heading word, floating sub-heading word, keyword heading word, organism supplementary concept word, protocol supplementary concept word, rare disease supplementary concept word, unique identifier, synonyms] | 1210801 |
| 17 | 8 or 9 or 10 or 11 or 12 or 13 or 14 or 15 or 16 | 3700982 |
| 18 | exp kidney failure/ | 194183 |
| 19 | (acute renal$ or acute kidney$).tw. | 60754 |
| 20 | ("acute renal failure" or "acute kidney disease" or "acute kidney injury" or "acute renal insufficiency").tw. | 55040 |
| 21 | 18 or 19 or 20 | 218952 |
| 22 | exp maternal mortality/ | 11087 |
| 23 | exp intensive care unit/ | 100402 |
| 24 | exp hypertension/ | 307825 |
| 25 | exp heart muscle ischemia/ | 0 |
| 26 | exp coronary artery disease/ | 71714 |
| 27 | exp heart failure/ | 139164 |
| 28 | exp cerebrovascular accident/ | 160607 |
| 29 | exp cardiovascular disease/ | 2627342 |
| 30 | exp lung embolism/ | 0 |
| 31 | exp cardiomyopathy/ | 104096 |
| 32 | exp vein thrombosis/ | 0 |
| 33 | "TECHNETIUM TC 99M".tw. | 639 |
| 34 | ("maternal outcomes" or "ITU" or "intensive care unit" or "critical care unit" or "CCU renal outcomes" or "progressive renal failure" or hypertension or "ischaemic heart disease").tw. | 547417 |
| 35 | (" IHD" or "coronary artery disease" or CAD or "coronary heart disease" or "myocardial infarction").tw. | 334885 |
| 36 | ("cardiovascular disease" or " CVD" or "acute coronary syndrome" or ACS or "heart failure" or "cardiac failure" or "left ventricular systolic dysfunction" or stroke).tw. | 638347 |
| 37 | ("cerebrovascular disease" or "cerebrovascular accident" or "CVA " or cardiomyopathy or "peripheral vascular disease").tw. | 108306 |
| 38 | ("PE" or "pulmonary embolism" or "DVT" or "deep-vein thrombosis").tw. | 99799 |
| 39 | ("deep vein thrombosis" or "deep vein thrombus" or "VTE" or "venous thromboembolism" or microalbuminuria or microalbumin).tw. | 50711 |
| 40 | 22 or 23 or 24 or 25 or 26 or 27 or 28 or 29 or 30 or 31 or 32 or 33 or 34 or 35 or 36 or 37 or 38 or 39 | 3238380 |
| 41 | 7 and 17 and 21 and 40 | 2364 |
| 42 | limit 41 to humans | 2253 |
| **Embase** **Search Strategy** | | |
| 1 | pregnancy/ | 645881 |
| 2 | conception/ | 15149 |
| 3 | childbirth/ | 23353 |
| 4 | fetus/ | 204313 |
| 5 | newborn/ | 572873 |
| 6 | (pregnan$ or gestation$ or (new adj5 born) or labo$ or fetal$).tw. | 2007363 |
| 7 | 1 or 2 or 3 or 4 or 5 or 6 | 2633323 |
| 8 | clinical trial/ | 1036129 |
| 9 | exp cohort analysis/ | 853369 |
| 10 | exp case control study/ | 207127 |
| 11 | exp prospective study/ | 772285 |
| 12 | retrospective study/ | 1259698 |
| 13 | exp longitudinal study/ | 173949 |
| 14 | exp follow up/ | 1850567 |
| 15 | (cohort$ or (case$ adj5 control$) or (longitudinal$ adj5 stud$) or (follow$ adj5 stud$)).tw. | 1894943 |
| 16 | (prospective$ adj5 stud$).mp. or (retrospective$ adj5 stud$).tw. [mp=title, abstract, heading word, drug trade name, original title, device manufacturer, drug manufacturer, device trade name, keyword heading word, floating subheading word, candidate term word] | 1683841 |
| 17 | 8 or 9 or 10 or 11 or 12 or 13 or 14 or 15 or 16 | 5652966 |
| 18 | exp kidney failure/ | 434937 |
| 19 | (acute renal$ or acute kidney$).tw. | 92823 |
| 20 | ("acute renal failure" or "acute kidney disease" or "acute kidney injury" or "acute renal insufficiency").tw. | 84631 |
| 21 | 18 or 19 or 20 | 450192 |
| 22 | exp maternal mortality/ | 22750 |
| 23 | exp intensive care unit/ | 249634 |
| 24 | exp hypertension/ | 838975 |
| 25 | exp heart muscle ischemia/ | 97862 |
| 26 | exp coronary artery disease/ | 364411 |
| 27 | exp heart failure/ | 579722 |
| 28 | exp cerebrovascular accident/ | 267415 |
| 29 | exp cardiovascular disease/ | 4575393 |
| 30 | exp lung embolism/ | 107724 |
| 31 | exp cardiomyopathy/ | 161461 |
| 32 | exp vein thrombosis/ | 146167 |
| 33 | "TECHNETIUM TC 99M".tw. | 698 |
| 34 | ("maternal outcomes" or "ITU" or "intensive care unit" or "critical care unit" or "CCU renal outcomes" or "progressive renal failure" or hypertension or "ischaemic heart disease").tw. | 827710 |
| 35 | (" IHD" or "coronary artery disease" or CAD or "coronary heart disease" or "myocardial infarction").tw. | 499377 |
| 36 | ("cardiovascular disease" or " CVD" or "acute coronary syndrome" or ACS or "heart failure" or "cardiac failure" or "left ventricular systolic dysfunction" or stroke).tw. | 1007443 |
| 37 | ("cerebrovascular disease" or "cerebrovascular accident" or "CVA " or cardiomyopathy or "peripheral vascular disease").tw. | 173817 |
| 38 | ("PE" or "pulmonary embolism" or "DVT" or "deep-vein thrombosis").tw. | 138653 |
| 39 | ("deep vein thrombosis" or "deep vein thrombus" or "VTE" or "venous thromboembolism" or microalbuminuria or microalbumin).tw. | 81934 |
| 40 | 22 or 23 or 24 or 25 or 26 or 27 or 28 or 29 or 30 or 31 or 32 or 33 or 34 or 35 or 36 or 37 or 38 or 39 | 5107712 |
| 41 | 7 and 17 and 21 and 40 | 9657 |
| 42 | limit 41 to humans | 9411 |
| 43 | pregnancy/ | 645881 |

**Table S2.** Outcome of interest and definitions used in this study

| **Outcome** | **Definition** |
| --- | --- |
| Maternal mortality^1^ | Defined as the death of a woman during pregnancy or childbirth or within 42 days of termination of the pregnancy, irrespective of duration and site of the pregnancy. |
| Hypertension^2^ | Hypertension is defined as a pressure in the blood vessels of 140/90mmHg or higher |
| Gestational hypertension^3^ | New onset hypertension in pregnancy with a blood pressure of 140/90mmHg or higher with the absence of proteinuria. |
| Heart failure^4^ | A clinical syndrome with symptoms and/or signs caused by a structural and/or functional cardiac abnormality with elevated natriuretic peptides. There may also be objective evidence of pulmonary or systemic congestion. |
| Stroke^5^ | A clinical syndrome of presumed vascular origin characterised by rapidly developing signs of focal or global disturbance of cerebral functions which lasts longer than 24 hours or leads to death. |
| Thrombotic microangiopathy^6^ | A clinical syndrome due to a pathological lesion that occurs due to endothelial injury. It is a heterogeneous group of disorders that is typically characterised by microangiopathic haemolytic anaemia, thrombocytopenia and end-organ ischemia. It can also be renally limited with no systemic features. |
| Partial renal recovery | It is defined as suboptimal recovery in renal function within 3 months. |
| Renal insufficiency | For the purpose of this study, it is defined as a persistent or worsening of renal function within 3 months of an episode of AKI in pregnancy. |
| Chronic kidney disease^7^ | It is defined as a reduction in renal function that is persistent beyond 3 months. |
| Renal replacement therapy or Dialysis | For the purpose of this study, we have excluded renal transplantation. It refers to temporary or permanent dialysis to replace renal function. |
| End-stage renal failure | Refers to CKD stage 5 as per KDIGO or requiring renal replacement therapy. |
| Intensive care unit admissions | Admission for any length of time to the intensive or high dependency care unit, depending on the setting of the study. |

**Table S3.** Study design and participant characteristics.

| **Study ID** | **Study Design; Country; Year** | **Total No. of Women (AKI/no AKI)** | **Mean Age (AKI vs no AKI)** | **Participant Selection Criteria** | **Duration of follow-up** |
| --- | --- | --- | --- | --- | --- |
| Beers 2020^8^ | Retrospective cohort study, United States of America, 2020 | 48,350,431 (34,001 vs 48,316,430) | 28.87 vs 27.74 | Pregnancy-related hospitalisations between 2005 and 2015.  AKI was defined using ICD codes.  Outcome: maternal mortality.  Adjusted for comorbidities (diabetes mellitus, hypertension, anaemia, chronic pulmonary disease, congestive heart failure, hypothyroidism, electrolyte imbalance, chronic liver disease, obesity, renal failure, AIDS, metastatic cancer, rheumatoid arthritis, psychosis, alcohol abuse, drug abuse), median household income, primary payer (Medicare/Medicaid, private insurance, self-pay, or no charge), admission type, and hospital-level characteristics such as hospital bed size (small, medium, and large), region (Northeast, Midwest or North Central, South, and West), and teaching status. | 20 days from hospitalisation |
| Bouaziz 2013^9^ | Retrospective cohort, Tunisia, 1995-2011 | 550 (313 vs 237) | 30.5 vs 30.8 | Admission to ICU without baseline renal disease or hypertension, end point unclear.  AKI was defined by serum creatinine level >0.8 mg/dL.  Outcomes: chronic kidney disease, dialysis, ICU admission, maternal mortality. | 17 days from hospitalisation |
| Frances Conti-Ramsden 2019^1^ | Prospective multicentre cohort study, South Africa, 2019 | 1547 (237 vs 1310) | 28.2 vs 27.5 | Women with a diagnosis of pre-eclampsia between January 2015 – May 2016.  AKI was defined as per the KDIGO criteria.  Outcomes: ICU admission, maternal mortality, stroke. | 1 year |
| Gama 2021^2^ | Retrospective case control study, United Kingdom, 2021 | 864 (288 vs 576) | 32.7 vs 32.3 | Women aged between 18 and 55 years receiving obstetric care between November 2016 and October 2018.  AKI was defined using KDIGO guidelines.  Outcomes: chronic kidney disease, renal recovery.  Adjusted for age, ethnicity, gestational age, parity, Caesarean delivery, hypertension (pregnancy-induced, chronic and pre-eclampsia), diabetes (pre-existing and gestational diabetes), obesity (BMI>30 kg/m^2^), CKD (pre-pregnancy KDIGO criteria), | 3 months |
| Gul 2004^3^ | Prospective Cohort Study, Turkey, 2004 | 132 (20 vs 112) | 27.9 vs 27.8 | Pregnant women with hypertensive disorders admitted between January 15, 2002 and September 15, 2003.  AKI was defined as creatinine level > 1.2 mg/dL and/or oliguria <400 mL/24 hr. The cases were divided into three groups on the basis of the highest creatinine level recorded during hospitalisation: creatinine <1.2, creatinine 1.2 to 2.0 mg/dL, and creatinine 2.0 mg/dL.  Outcome: dialysis. | 23 days from hospitalisation |
| Hildebrand 2015^4^ | Retrospective cohort, Canada, 1997-2011 | 1,918,789 (188 vs  1,918,601) | 32 vs 30 | Linked health care databases which capture all hospital childbirths in the province, defined as delivery of an infant at a gestational age of >20 weeks.  AKI patients were identified using the ICD codes for receipt of at least one acute dialysis treatment during pregnancy or within 12 weeks postpartum.  Outcomes: gestational hypertension, heart failure, maternal mortality, peripheral vascular disease, renal replacement therapy, thrombotic microangiopathy | 3 months from delivery |
| Liu 2015^5^ | Retrospective case control, China, 2004-2013 | 38 (22 vs 16) | 30.9 | Women admitted between July 2004 to February 2013.  AKI was defined as an absolute increase in the serum creatinine (Scr) level of 0.3 mg/dL (26.5 μmol/L), an increase in the Scr level of 50% (1.5 times the baseline value) or a urinary volume of <0.5 mL/kg/h for more than six hours. Since an Scr level of >70 μmol/L usually indicates renal dysfunction in pregnant women, a Scr level of >70 μmol/L prior to the pregnancy endpoint was chosen as a threshold for the diagnosis of AKI.  Outcome: hypertension. | 40 days from hospitalisation |
| Liu 2019^6^ | Retrospective multicentre cohort study, China, 2019 | 11,073 (809 vs 10,264) | 29.7 (community AKI), 30.2 (hospital AKI) and 30.3 (no AKI) | Women of childbearing age who had at least two serum creatinine (SCr) tests within any 7-day window during hospitalisation from January 1, 2013 to December 31, 2015. Excluded patients with end-stage renal disease, receiving maintenance dialysis or renal transplantation.  AKI was defined as per the KDIGO criteria.  Outcomes: dialysis, heart failure, hypertension, ICU admission, maternal mortality, peripheral vascular disease, thrombotic microangiopathy.  Adjusted for age, baseline creatinine, length of stay in hospital, division, hospital and comorbidities (chronic kidney disease, convulsion, chronic obstructive pulmonary disease, diabetes mellitus, diarrhoea, gastrointestinal bleeding, peptic ulcer disease, heart failure, haematological tumour, hypertension, liver disease, pulmonary infection, myocardial infarction, glomerulonephritis, pregnancy-induced hypertension syndrome, acute fatty liver, sepsis, shock, systemic lupus erythematosus, stroke, thrombotic microangiopathy, trauma, hyperlipidaemias). | 30 days from hospitalisation |
| Mjahed 2004^7^ | Prospective cohort study, Morocco, 1993-2000 | 178 (46 vs 132) | 28.4 vs 26.4 | Women with eclampsia admitted to intensive care between January 1, 1993 and December 31, 2000.  AKI was defined by a serum creatinine concentration >140 mmol/L at the time of admission to the intensive care unit without preexisting renal disease.  Outcomes: chronic kidney disease, maternal mortality. | 15 days |
| Muzammil 2021^10^ | Prospective cohort study, Pakistan, 2021 | 53 (13 vs 40) | 27.3 AKI stage 1, 27.1 AKI stage 2 and 26.9 AKI stage 3 vs 26.3 (non-AKI) | Women with preeclampsia admitted between 5th June 2020 to 5th June 2021 who were diagnosed with preeclampsia.  AKI was defined as per the KDIGO criteria.  Outcomes: ICU admission, maternal mortality, stroke. | 1 year |
| Rodríguez-Benitez 2021^11^ | Retrospective cohort study, Spain, 2021 | 303 (75 vs 228) | 35.09 vs 33.56 | A hospital-based cohort of patients with severe preeclampsia treated at a tertiary centre between January 2007 and December 2018.  AKI was defined as per the KDIGO group.  Outcomes: gestational hypertension, hypertension, peripheral vascular disease, prolonged renal insufficiency, thrombotic microangiopathy. | 12 weeks from delivery |
| Shah 2020^12^ | Retrospective cohort study, United States of America, 2020 | 42,223,175 (32,385  vs  42,190,790) | 28 vs 26.9 | Identified delivery hospitalisations from the NIS database that occurred in women during pregnancy from January 1, 2006 to September 30, 2015, in the United States.  AKI was defined using ICD-9 codes.  Outcomes: dialysis, heart failure, stroke.  Adjusted for calendar year, age, race/ethnicity, insurance, residential income quartile, delivery, admission type, hospital characteristics (bed size, location, region), Elixhauser mortality score, diabetes, and eclampsia/pre-eclampsia. | 6 days from delivery |
| Sharma 2021^13^ | Prospective cohort study, India, 2021` | 104 (26 vs 78) | 27.42 vs 28.4 | Pregnant women with preeclampsia admitted between January 2019 to January 2020. Excluded obstetric complications e.g. septic abortion, abruptio placentae, uterine haemorrhage, intrauterine foetal death, and puerperal sepsis.  AKI was defined as per the KDIGO criteria.  Outcomes: chronic kidney disease, dialysis, maternal mortality, prolonged renal insufficiency. | 3 months from hospitalisation |
| Wang 2020^14^ | Retrospective case control study, China, 2020 | 1253 (625 vs 628) | 31.8 vs 30.9 | Gestational age of >20 weeks, an available baseline serum creatinine concentration and complete clinical data.  AKI was defined as a 50% increase in the serum creatinine concentration from baseline and a concentration of >70.72 mmol/L. Moderate to severe AKI was defined as a serum creatinine concentration of >124 mmol/L.  Outcomes: renal replacement therapy, maternal mortality.  Adjustment for age, comorbidities, leukocyte count, transaminase concentrations, total bilirubin concentration, coagulation parameters and serum albumin concentration. | NA |
| Wang 2021^15^ | Retrospective cohort study, China, 2021 | 110 (65 vs 45) | 31.26 vs 30.22 | Pregnant patients diagnosed with HELLP syndrome.  AKI was defined using the KDIGO classification.  Outcome: maternal mortality, prolonged renal insufficiency, renal replacement therapy. | 1 year |
| William Cooke 2018^16^ | Prospective cohort study, Malawi, 2018 | 302 (26 vs 276) | 27 vs 25 | Women greater than 20 weeks gestation or less than six weeks postpartum within 48 hours of admission with risk factors for AKI: gestational hypertension, preeclampsia, eclampsia, antepartum haemorrhage, postpartum haemorrhage, sepsis, heart failure and renal failure. Baseline clinical data were recorded, and screening serum creatinine was measured to determine the presence/absence of kidney disease.  AKI was staged according to the KDIGO criteria.  Outcomes: maternal mortality, prolonged renal insufficiency. | 3 months |
| Ye 2019^17^ | Retrospective cohort study, China, 2019 | 108 (52 vs 56) | 31.19 vs 31.39 | Patients who received intensive postpartum care because of serious complications.  AKI was classified as per the KDOQI criteria.  Outcome: maternal mortality, prolonged renal insufficiency, renal replacement therapy. | 2 years |

AIDS, acquired immune deficiency syndrome. AKI, acute kidney injury. HELLP, haemolysis, elevated liver enzymes, low platelet count. ICD, International Classification of Diseases. ICU, Intensive Care Unit. KDIGO, Kidney Disease Improving Global Outcomes. KDOQI, Kidney Disease Outcomes Quality Initiative.

**Table S4.** Study quality assessment

| **Study ID** | **Representative of the exposed cohort** | **Selection of the non-exposed cohort** | **Ascertainment of exposure** | **Demonstration that outcome of interest was not present at the start of study** | **Comparability of cohort** | **Assessment of outcome** | **Follow-up duration to capture outcomes** | **Adequacy of follow-up** | **Total** |
| --- | --- | --- | --- | --- | --- | --- | --- | --- | --- |
| Beers 2020 | * | * | * | * | * | * | * | * | **8** |
| Bouaziz 2013 |  | ***** | ***** | ***** |  | ***** | ***** |  | **5** |
| Frances 2019 |  |  | ***** | ***** | ***** |  | ***** |  | **4** |
| Gama 2021 |  | ***** | ***** | ***** | ***** | ***** |  |  | **5** |
| Gul 2004 |  |  | ***** | ***** |  | ***** | ***** | ***** | **5** |
| Hildebrand 2015 | ***** | ***** |  | ***** | ***** | ***** | ***** | ***** | **7** |
| Liu 2015 | ***** |  | ***** | ***** |  |  |  |  | **3** |
| Liu 2019 | ***** | ***** | ***** | ***** | ***** | ***** | ***** |  | **7** |
| Mjahed 2004 |  | ***** | ***** | ***** | ***** | ***** |  |  | **5** |
| Muzammil 2021 |  |  | ***** | ***** |  | ***** |  |  | **3** |
| Rodríguez-Benitez 2021 | ***** | ***** | ***** | ***** | ***** | ***** | ***** | ***** | **8** |
| Shah 2020 | * | * | * |  | * | * | * | * | **7** |
| Sharma 2021 | ***** | ***** | ***** | ***** | ***** | ***** |  |  | **6** |
| Wang 2020 |  |  | ***** | ***** |  | ***** | ***** |  | **4** |
| Wang 2021 |  | ***** | ***** | ***** | ***** | ***** | ***** | ***** | **6** |
| William 2018 | ***** | ***** | ***** | ***** |  | ***** |  |  | **5** |
| Ye 2019 |  | ***** | ***** | ***** | ***** | ***** |  |  | **5** |

*Green colour indicates a low risk of bias; yellow shading indicates a moderate risk of bias and red shading indicates a high risk of bias

**Table S5.** Subgroup analysis of outcomes of AKI in pregnancy based on the definition.

| **Outcome** | **Defined according to KDIGO** | **Defined by arbitrary serum creatinine level** |
| --- | --- | --- |
| **Maternal mortality** | 6.10 [2.96, 12.57] | 8.55 [4.12, 17.73] |
| **Composite adverse renal outcome (CKD/ RRT)** | 21.15 [4.91, 91.06] | 12.69 [2.24, 72.01] |

Values are represented as odds ratio [95% confidence interval].

CKD, chronic kidney disease. RRT, renal replacement therapy.

**Table S6.** Subgroup analysis of outcomes of AKI in pregnancy based on presence of pre-eclampsia.

| **Outcome** | **Pre-eclampsia** | **No pre-eclampsia** |
| --- | --- | --- |
| **Composite adverse renal outcome (CKD/ RRT)** | 12.83 [3.35, 49.13] | 549.42 [0.93, 326294.95] |
| **Dialysis** | 38.22 [7.13, 204.80] | 25.56 [6.38, 102.38] |
| **Gestational hypertension** | 0.59 [0.31, 1.13] | 1.79 [0.80, 4.05] |
| **Hypertension** | 0.90 [0.49, 1.66] | 1.33 [0.25, 6.95] |
| **ICU admission** | 2.73 [2.07, 3.60] | 6.03 [4.62, 7.86] |
| **Maternal mortality** | 5.63 [3.45, 9.18] | 35.78 [6.32, 202.44] |
| **Thrombotic microangiopathy** | 6.87 [3.25, 14.51] | 11.21 [0.69, 182.29] |

Values are represented as odds ratio [95% confidence interval].

CKD, chronic kidney disease. RRT, renal replacement therapy.

**Table S7.** Subgroup analysis based on study location

| **Outcomes** | **Asia** | **Africa** | **Europe** | **North America** | **South America** |
| --- | --- | --- | --- | --- | --- |
| **Maternal Mortality** | 8.80 [4.17, 18.57] | 4.15 [2.42, 7.11] | - | 18.87 [2.02, 175.90] | - |
| **Hypertension** | 1.33 [0.25, 6.95] | - | 0.90 [0.49, 1.66] | - | - |
| **Gestational Hypertension** | - | - | 1.79 [0.80, 4.05] | 1.79 [0.80, 4.05] | - |
| **ICU admission** | 5.84 [4.39, 7.76] | 2.71 [2.04, 3.60] | - | - | - |
| **Heart Failure** | 2.96 [2.11, 4.15] | - | - | 61.61 [13.27, 285.99] | - |
| **Stroke** | 3.25 [0.19, 55.98] | 16.78 [1.74, 162.03] | - | 91.92 [81.65, 103.49] | - |
| **Thrombotic Microangiopathy** | 3.33 [1.35, 8.19] | - | 6.87 [3.25, 14.51] | 35.98 [23.61, 54.85] | - |
| **Partial renal recovery/ renal insufficiency** | 24.39 [4.49, 132.58] | 110.60 [5.77, 2119.82] | 50.04 [2.82, 887.29] | - | - |
| **Dialysis** | 26.52 [7.55, 93.17] | 14.82 [0.86, 255.91] | 125.00 [6.76, 2312.67] | 8234686.00 [514933.99, 131686884.87] | - |
| **Composite adverse renal outcome (CKD/ RRT)** | 25.78 [6.06, 109.62] | 5.64 [0.62, 51.40] | 22.37 [1.23, 405.94] | 158562.11 [9022.68, 2786527.95] | - |

Values are represented as odds ratio [95% confidence interval].

ICU, intensive care unit. CKD, chronic kidney disease. RRT, renal replacement therapy.

(A)

**
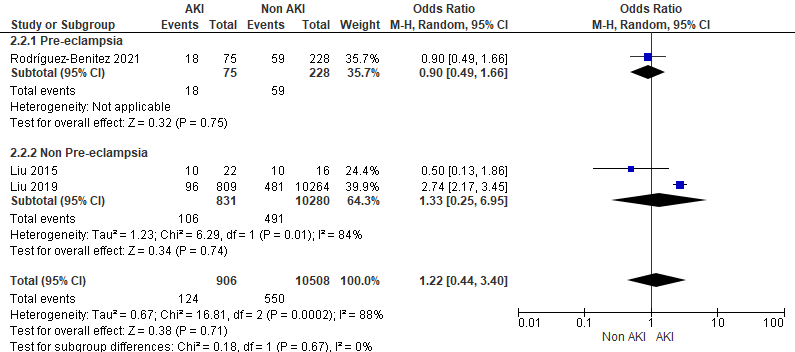
**

(B)

**
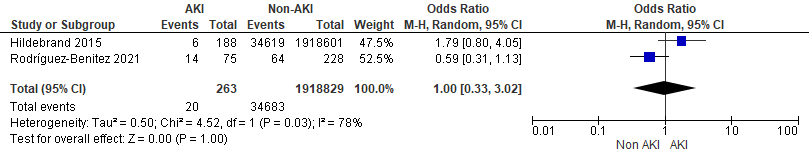
**

**Figure S1.** Risk of (A) hypertension or (B) gestational hypertension with acute kidney injury in pregnancy.

**Supplementary References**

1. Conti-Ramsden FI, Nathan HL, De greeff A, et al. Pregnancy-Related Acute Kidney Injury in Preeclampsia. Hypertension 2019. DOI: 10.1161/HYPERTENSIONAHA.119.13089.

2. Gama RM, Clark K, Bhaduri M, et al. Acute kidney injury e-alerts in pregnancy: rates, recognition and recovery. Nephrol Dial Transplant 2020. DOI: 10.1093/ndt/gfaa217.

3. Gul A, Aslan H, Cebeci A, et al. Maternal and fetal outcomes in HELLP syndrome complicated with acute renal failure. Ren Fail 2004. DOI: 10.1081/jdi-200031750.

4. Hildebrand AM, Liu K, Shariff SZ, et al. Characteristics and Outcomes of AKI Treated with Dialysis during Pregnancy and the Postpartum Period. J Am Soc Nephrol 2015. DOI: 10.1681/ASN.2014100954.

5. Liu Y, Bao H, Jiang Z, et al. Pregnancy-related Acute Kidney Injury and a Review of the Literature in China. Intern Med 2015. DOI: 10.2169/internalmedicine.54.3870.

6. Liu D, He W, Li Y, et al. Epidemiology of acute kidney injury in hospitalized pregnant women in China. BMC Nephrology 2019. DOI: 10.1186/s12882-019-1255-8.

7. Mjahed K, Alaoui SY and Barrou L. Acute renal failure during eclampsia: incidence risks factors and outcome in intensive care unit. Ren Fail 2004. DOI: 10.1081/jdi-120039518.

8. Beers K, Wen HH, Saha A, et al. Racial and Ethnic Disparities in Pregnancy-Related Acute Kidney Injury. Kidney360 2020. DOI: 10.34067/KID.0000102019.

9. Bouaziz M, Chaari A, Turki O, et al. Acute renal failure and pregnancy: a seventeen-year experience of a Tunisian intensive care unit. Ren Fail 2013. DOI: 10.3109/0886022X.2013.819767.

10. Muzammil M, Qureshi AI and Imran H. Risk Factors and Prognosis of Acute Kidney Injury in Pre-Eclampsia 2021.

11. Rodríguez-Benitez P, Aracil Moreno I, Oliver Barrecheguren C, et al. Maternal-Perinatal Variables in Patients with Severe Preeclampsia Who Develop Acute Kidney Injury. J Clin Med 2021. DOI: 10.3390/jcm10235629.

12. Shah S, Meganathan K, Christianson AL, et al. Pregnancy-Related Acute Kidney Injury in the United States: Clinical Outcomes and Health Care Utilization. Am J Nephrol 2020. DOI: 10.1159/000505894.

13. Sharma M, Mazumder MA, Alam S, et al. Characteristics, maternal and neonatal outcomes of acute kidney injury in preeclampsia: A prospective, single-center study. Clin Nephrol 2021. DOI: 10.5414/CN110447.

14. Wang B, Jiang Q and Wu X. Association of D-dimers with acute kidney injury in pregnant women: a retrospective study. J Int Med Res 2020. DOI: 10.1177/0300060520966899.

15. Wang L, Tang D, Zhao H, et al. Evaluation of Risk and Prognosis Factors of Acute Kidney Injury in Patients With HELLP Syndrome During Pregnancy. Front Physiol 2021. DOI: 10.3389/fphys.2021.650826.

16. Cooke WR, Hemmilä UK, Craik AL, et al. Incidence, aetiology and outcomes of obstetric-related acute kidney injury in Malawi: a prospective observational study. BMC Nephrology 2018. DOI: 10.1186/s12882-018-0824-6.

17. Ye W, Shu H, Yu Y, et al. Acute kidney injury in patients with HELLP syndrome. Int Urol Nephrol 2019. DOI: 10.1007/s11255-019-02111-7.
